# The central role of symptom severity and associated characteristics for functional impairment in misophonia

**DOI:** 10.3389/fpsyt.2023.1112472

**Published:** 2023-03-28

**Authors:** Anne Möllmann, Nina Heinrichs, Lisa Illies, Nadine Potthast, Hanna Kley

**Affiliations:** Department of Psychology, Faculty of Psychology and Sports Science, Bielefeld University, Bielefeld, Germany

**Keywords:** misophonia, functional impairment, emotion regulation, decreased sound tolerance, misophonic reactions

## Abstract

Misophonia is characterized by a preoccupation with and strong emotional and behavioral reactions to certain triggers, mostly sounds related to eating and breathing. We applied functional impairment due to misophonic symptoms as a central criterion to investigate differences between clinical misophonia and normative decreased sound tolerance in a large non-random sample of *n* = 1,881 individuals from an online survey. We assessed the frequency of self-reported misophonia symptoms across various symptom measures, compared severity, triggers and emotional reactions, general psychopathology, interpersonal emotion regulation, and quality of life between both groups with and without functional impairing misophonia. Individuals with functional impairment due to misophonia (*n* = 839) revealed significantly higher general psychopathology symptoms, lower interpersonal emotion regulation skills, and lower quality of life than individuals without impairment (*n* = 1,042). Anxious/distressed and annoyed reactions to triggers were experienced more frequently compared to emotional reactions of disgust and sadness or depression in both groups. Overall, the group differences were primarily quantitative in nature. We discuss practical implications regarding classification and treatment and provide cutoffs for each symptom measure derived from group assignment for functional impairment.

## 1. Introduction

Misophonia is described as a condition marked by negative reactions toward primarily oral and nasal auditory stimuli, such as smacking or slurping. These are usually conceptualized as “triggers” as they are responsible for the sequelae of subsequent intense emotional (e.g., irritation, anxiety, disgust, and tension), physiological (e.g., increased heart rate), cognitive (e.g., internalizing or externalizing appraisal of own reaction), and behavioral (e.g., avoid the stimuli) reactions (1). Misophonia is currently not included in the International Classification of Diseases (ICD-11) (2) or Diagnostic and Statistical Manual of Mental Disorders (DSM-5-TR) (3) although most research demonstrates significant symptom presentation which seems unrelated to hearing levels or perceptual abilities (1, 4, 5).

The existing consensus definition on misophonia includes a general description (1) but where and how to define a clinical threshold for the specific features and their combination is a challenge still to be mastered. Accordingly, estimates on how often misophonia occurs [e.g., reported here, (6–17)] are characterized by a considerable heterogeneity regarding measures and suggested cutoffs. For example, in the cited studies, ten different measures in six different languages were used to assess misophonia, mostly using self-reports such as the Amsterdam Misophonia Scale (A-MISO-S) (18), the Misophonia Questionnaire (MQ) (16), or one item only (7). Some studies included a diagnostic interview on mental disorders and/or misophonia as a disorder (14, 18, 19), which includes functional impairment. The heterogeneity led to frequency estimates between 5 and 49% across studies, with sample sizes ranging from 94 (14) and 2,519 (9), including two samples representative of the general population (9, 10). For estimates based on self-report, 5% (9) and 7.3% (15) may be the best evidence currently available, for the first study because of the large representative sample, but the estimate is based on one item only (item 18 = the MQ severity scale); for the latter because of the sample size and the use of the Duke-Vanderbilt Misophonia Screening Questionnaire (DVMSQ), which includes impairment in its screening algorithm. Still, Swedo et al. (1) concluded in their Delphi study that a prevalence estimate would be premature.

The higher estimates [e.g., about 35–50% in a help-seeking or student sample (8, 11)] usually include a mild symptoms group as suggested by the developers of the A-MISO-S (18). However, it is unclear if mild symptoms correspond to a clinical level of a mental disorder including sufficient functional impairment or suffering. This is somewhat implied as five groups can be identified with the A-MISO-S and only one group is described as “no symptoms/subclinical.” Questions on the extent of distress and functional impairment are included; however, it is not required to indicate impairment or suffering to score in the clinical groups. The MQ in contrast derives the clinical group assignment from one item only, leading to estimates for clinical symptoms of 16.6–19.9% in college students (16, 20).

Misophonia symptoms have been reported to be associated with symptoms of other mental disorders and lower quality of life in clinical as well as student samples. In their large clinical sample, Jager et al. (19) reported mild symptoms of anxiety and depression and lower quality of life. The comorbidity rate was 28%, with major depressive, attention deficit (hyperactivity), and obsessive–compulsive disorder as the most common comorbid disorders. The prevalence of these disorders was elevated in the misophonia sample compared to the general population. Obsessive–compulsive personality traits were also very common in the clinical sample (23.8%). Accordingly, Wu et al. (16) reported medium significant correlations of misophonia symptoms and anxiety, obsessive–compulsive, and depressive symptoms in their student sample. Emotion regulation difficulties were reported to be associated with misophonia symptoms, even beyond the associations with depressive and anxiety symptoms (21, 22). As both, the experienced emotions toward certain sounds and the appraisals of the emotional reaction (externalizing: blaming others; internalizing: blaming oneself), differ in individuals with versus without misophonia (23), a closer investigation of emotion regulation processes appears warranted. Many individuals report misophonic symptoms to occur with family members and/or significant others but not, or at least not to the same extent, to unfamiliar others. We were therefore interested to evaluate if there is any relation to interpersonal aspects that may function as a discriminating factor between individuals with or without misophonic disorder.

In the present study, we examined responses from an online survey advertised through different media [(online) newspaper, social media, and mailing lists] to recruit individuals who are affected as well as unaffected by misophonia. The study had three aims: First, to assess the frequency of self-reported misophonia symptoms and compare the scoring across various common symptom measures in a large sample (*n* = 1,881). Second, to perform a clinical group assignment based on functional impairment due to misophonia symptoms and examine differences between these groups regarding symptoms, associated characteristics (e.g., triggers), general psychopathology, interpersonal emotion regulation, and quality of life. Third, to provide cutoffs for existing symptom measures to differentiate functional impairing from non-impairing misophonia based on the same measure of functional impairment for each symptom measure.

## 2. Materials and methods

### 2.1. Participants

Two thousand one hundred and fifty individuals started the online survey. The data of *n* = 1,881 individuals were included in the analyses^1^. Reasons for exclusion of *n* = 269 participants were: aged under 18 (*n* = 23); participation terminated prior to main measure for the current study (*n* = 246). The mean age of the participants was *M* = 33.10 years (*SD* = 11.29; range: 18–77), 1,556 individuals (82.7%) self-identified as female (male: 16.9%, non-binary or trans: 0.4%). Approximately 71% of the participants (*n* = 1,326) were living in a relationship (cohabitating) or were married. Most participants were well educated (high school degree: 37.7%; college degree: 45.0%). The first question of the survey was introduced after a brief description of misophonia and requested a self-assignment on how much the description applies the participants (translated description: “Misophonia is an increased sensitivity to specific sounds (e.g., eating sounds, pen clicking, and breathing sounds), and/or an increased sensitivity to movement (e.g., bobbing with a leg) which regularly leads to extreme emotional reactions (e.g., irritability, anger, and disgust)” - “This definition describes myself: 0 “not at all” to 100 “entirely”). 87.5% (*n* = 1,646) of the individuals indicated that the description applied to them with scores ≥50.

### 2.2. Procedure

The online survey study was approved by the local institutional review board of the University of Bielefeld. The survey link was provided on the psychotherapy outpatient clinic website, *via* private social networks, and e-mail distribution lists of psychology students and individuals interested in misophonia research. Several national (online) media became aware of the study and included the link in their articles on misophonia. Participation was possible between September 2020 and May 2021. Pilot trials indicated a duration of 30–45 min to complete the survey. All participants provided informed consent prior to participation. Participants were able to take part in a lottery of ten gift cards of 10€ each, students from the local university received course credit.

### 2.3. Materials

#### 2.3.1. Misophonia measures

Most of the misophonia measures described below include several items on distress due to misophonia and only single items on functional impairments caused by the symptoms. However, the respective cutoffs for clinical misophonia can be exceeded without having indicated functional impairments on the items. We were specifically interested in impairments in the main areas of functioning due to misophonia, as this is one important aspect in the diagnostic criteria of mental disorders according to DSM-5 and ICD-11. We assessed functional impairments due to misophonia symptoms with the Work and Social Adjustment Scale (WSAS), a five item self-report measure developed to assess “functional impairment attributable to an identified problem” (pp. 461), beyond both disorder specific syndromes and the potential distress the syndromes may cause (24). The items (e.g., “Because of my <<misophonia symptoms>>, my ability to work is impaired”) are rated from 0 *no impairment* to 8 *very severe impairment*. In the current study, 170 participants skipped (only) the WSAS in the survey. These participants had indicated an impairment of 0 in the introductory question at the beginning of the survey. Thus, their WSAS score was set to 0.

Misophonia symptoms and related aspects were assessed with eight different self-report questionnaires, with higher sum scores indicating higher symptom severity. The measures were translated and back-translated from the original language (Dutch or English) to German according to a standardized translation and back-translation protocol. More detailed information on the translation process are presented in the electronic Supplementary material ESM1.

Three of these measures come from the Amsterdam based research group, the 14-item Misophonia Screening List [MSL (25), German translation (26)], the 7-item Amsterdam Misophonia Scale [A-MISO-S (18), German translation (27)] and the 12-item revised version of the A-MISO-S [AMISOS-R (28), German translation (29)]. In each instrument, approximately half of the items refer to the main symptoms (e.g., “How much of your time is occupied by misophonic sounds?” or “How intense is your feeling of irritability/anger when you hear these sounds?”) and the other half to impairments in functioning, distress, suffering and/or avoidance due to the symptoms. Regarding the emotional reactions and impulses, Schröder and colleagues focus on irritability, anger, aggressive impulses, and disgust. The A-MISO-S was adapted for misophonia from the Yale-Brown Obsessive–Compulsive Scale (YBOCS) (30), a gold standard measure to assess symptom severity of obsessive–compulsive disorder. Accordingly, aspects of resistance against and control over (obsessive, here: misophonia trigger related) thoughts are considered in the A-MISO-S and AMISOS-R items, respectively. In the AMISOS-R, the respondent is first asked to identify his*her individual trigger and emotional reaction (yes/no) and answer the subsequent items regarding this individual profile of the most disturbing trigger and typical emotion. There are slight but potentially significant differences between the measures, the reference period and the way misophonia triggers are defined: the A-MISO-S relates to the last 7 days, and counts any trigger (“sound, sight, touch, motion, etc.); the AMISOS-R relates to the last 3 days, and counts different sound triggers (e.g., eating sounds, nasal sounds, ambient noises); and the MSL does not relate to a time period, and speaks of “sounds people make.” Regarding the scoring, symptom severity in the A-MISO-S is indicated by a sum score of six out of seven items on a 5-point scale, in the AMISOS-R a total score is reflected by a sum of ten items on a 5-point scale for the respondents’ typical sound and emotion. According to Jager et al. (31), preliminary psychometric indices of the scale are good. For the MSL, psychometric indices and interpretation guidelines have not been published yet. See Table 1 for interpretation of the total scores of the measures. Internal consistencies in the current study were Cronbach’s α = 0.939 (MSL), α = 0.929 (AMISOS-R), and α = 0.862 (A-MISO-S).

**Table 1:** Misophonic symptom sum scores and their classification, based on existing cutoffs per measure, for the whole sample (*N* = 1,881).

		Sum score	Symptom severity					
			Mild^1^		Moderate	Severe	Extreme
				No to subclinical		Mild			
Questionnaire	Possible range	*M* (*SD*)	Classification of scores	*n* (%)	*n* (%)	*n* (%)	*n* (%)	*n* (%)
Amsterdam Misophonia Scale, A-MISO-S	0–24	10.13 (4.70)	No/subclinical: 0–4 Mild: 5–9 Moderate: 10–14 Severe: 15–19 Extreme: 20–24	261 (14.0)	524 (28.0)	764 (40.9)	291 (15.6)	30 (1.6)
Amsterdam Misophonia Scale Revised, AMISOS-R	0–40	21.43 (9.07)	Normal/subclinical: 0–10 Mild:11–20 Moderate severe: 21–30 Severe to extreme: 31–40	288 (15.4)	476 (25.4)	817 (43.6)	295 (15.7)	
Misophonia Assessment Questionnaire, MAQ	0–63	23.12 (15.58)	Mild: 0–21 Moderate: 22–42 Severe: 43–63	-	871 (47.1)	733 (39.6)	246 (13.3)	-
				Not clinically significant		Clinically significant		
Misophonia Questionnaire, MQ	0–68	29.09 (11.58)	Clinically significant^2^: item 18 > 6	986 (52.5)		892 (47.5)		
			item 18 > 6 & Mean_MQEB_ ≥ 2	1,192 (63.4)		687 (36.6%)		
Misophonia Screening List, MSL	0–56	28.53 (14.52)	No information	-	-	-	-	-

^1^The mild group is further differentiated in no/subclinical and mild (clinical) group according to the first two measures. ^2^The MQ differentiates between not-and clinically significant symptoms according to item 18 = the MQ Severity Scale. Rosenthal et al. suggest to combine a score > 6 on item 18 and mean item score of ≥ 2 on the MQ Emotions and behaviors subscale, Mean_MQEB_ (32).

The 18-item Misophonia Questionnaire [MQ (16), German translation (33)] assesses sensitivities to certain sounds in 7 items (same sounds as in AMISOS-R) and the frequency of misophonia-related emotional and behavioral reactions in 10 items (e.g., “Once you are aware of the sound (s), because of the sound (s), how often do you: Become sad or depressed?”). The sum score of the 17 items ranges from 0 to 68. Internal consistency of the MQ sum score was α = 0.879 in the current study. Additionally, one item (item 18 = MQ severity scale) assesses the self-rated severity of the sound sensitivity including related impairments or interferences in daily life [0 = *minimal* to 15 = *very severe*, scores above 6 indicating “clinically significant symptoms,” Wu et al. (16), p. 997].

The 21-item Misophonia Assessment Questionnaire [MAQ (34), German translation (35)] assesses a person’s emotional reactions and interferences with daily life caused by their so-called “sound issues” (e.g., “My sound issues make me feel guilty/unhappy/…”; “My sound issues impact my family relationships”) as well as some experiences with others’ reactions to the problem (e.g., “My sound issues have not been recognized as legitimate.”), with sum scores from 0 to 21 interpreted as *mild*, 22–42 as *moderate*, and 43–63 as *severe*. Internal consistency in the present study was α = 0.960.

The Misophonia Trigger List (MT) (36) contains 79 sounds which are to be rated regarding their trigger potential from 0 = “*does not apply*” to 4 = “*extremely applies*.” The sounds were derived from misophonia descriptions (37, 38) and extended by generally unpleasant sounds, such as mosquito buzzing, dentist’s drill, or toilet flushing.

We administered further misophonia measures (one targeted to assess diagnostic criteria; the other exploring other areas of impairment) in the present study, but this measure is intended to be used for a different purpose (a comparison for a clinical sample recruited in a different study) which is why the data are not reported here.

#### 2.3.2. Other measures

With one item each, we asked about other forms of diagnosed decreased sound tolerance, namely hyperacusis, tinnitus, and phonophobia, as well as any lifetime diagnosis of audiological or hearing disorders or any lifetime diagnosis of a mental disorder. Further, we asked for congenital sensory processing disorders or sensory hypersensitivity, experiencing Autonomous Sensory Meridian Response (ASMR), and synesthesia. The survey contained questions on demographic and health related information and questionnaires on the severity of general psychopathology according to diagnostic categories in the International Classification of Diseases (ICD-10), assessed with the 29-item ICD-10 Symptom Rating (ISR) (39), related general impairments (excluding misophonia-related impairments), assessed with the WSAS (24), quality of life, assessed with the Bielefelder Instrument for Quality of Life (BIFL) (40), and interpersonal emotion regulation, assessed with the Interpersonal Emotion Regulation Questionnaire (IERQ) (41).

### 2.4. Design and statistical analyses

The present study has a cross-sectional correlational design. We assigned participants to two groups according to their WSAS sum score of the five regular items and cutoffs, which we derived from the mean and standard deviation of a German WSAS validation study with depressed patients (i.e., clinical significant impairment from depression) (42): *no or mild impairment, associated with subclinical populations* = 0–8, *significant functional impairment* ≥ 9 (*moderate: 9–29; severe functional impairment* scores ≥30). Analyses were conducted with SPSS Version 26 and the KALPHA macro (43). We report descriptive statistics for the overall sample and chance-corrected agreement coefficient Krippendorff’s alpha per measure comparison. Group differences regarding measures on general psychopathology, interpersonal emotion regulation, and quality of life as well as trigger characteristics were analyzed with *t*-tests with Bonferroni-corrected alpha-levels and interpreted according to Cohen’s *d* effect sizes. Receiver operating characteristic (ROC) analyses and calculation of related coefficients were performed in RStudio with the packages *pROC* (44) and *bootLR* (45) to estimate the diagnostic capacity of the symptom measures quantified by the Area under the curve (AUC) to detect functionally impairing misophonia. Cutoff scores were derived based on the Youden Index (i.e., an index maximizing the sum of sensitivity and 1-specificity).

## 3. Results

### 3.1. Frequency of misophonic symptoms and functionally impairing misophonia in the overall sample

The sum scores and symptom severity levels of the misophonia measures for the overall sample are presented in Table 1. Applying the A-MISO-S and the AMISOS-R suggested cutoff scores to the present sample resulted in about 15% of the individuals without or with subclinical symptoms and about 85% with clinical, at least mild symptoms. According to the MAQ, “mild symptoms” is the lowest severity category, which applies to about 50% of the present sample, with the other 50% presenting with moderate to severe symptoms. Similarly, the MQ only differentiates clinically non-significant versus significant symptoms, which again resulted in about 50% of the sample for each category or—applying a stricter criterion (32)—about 60% in the clinically non-significant category, respectively. These frequencies are relatively high and indicate that many individuals may have participated because they self-referred to this condition.

Based on the binary categories per measure (c.f. Table 1; i.e., “mild” for the lowest two categories of the A-MISO-S and AMISOS-R and the lowest category of the MAQ and “moderate to extreme” for the remaining categories; similarly for the MQ item with categories “not” versus “clinically significant”), Table 2 presents the concordance rates and the chance-corrected agreement coefficient Krippendorff’s α between each two measures. Concordance rates ranged between 82 and 88% for the category “mild” and 72–85% for the category “moderate to extreme.” The chance-corrected agreement coefficients Krippendorff’s αs (46) ranged between 0.57 and 0.66.

**Table 2:** Concordance rates and chance-corrected agreement coefficient based on binary categorization per measure.

Measures		AMISOS-R		MAQ		MQ		WSAS - Misophonia	
		Mild	Moderate-extreme^1^	Mild	Moderate-extreme^1^	Not clinically significant	Clinically significant^1^	No functional impairment	Functional impairment
A-MISO-S	Mild	81.6%	15.1%	84.6%	15.4%	87.5%	12.5%	66.7%	11.4%
	Moderate-extreme^1^	18.4%	84.9%	19.6%	80.4%	27.1%	72.9%	33.3%	88.6%
	Krippendorff’s α [95%-CI]^2^	0.66 [0.63; 0.70]		0.64 [0.60; 0.67]		0.58 [0.54; 0.62]		0.53 [0.49; 0.57]	
AMISOS-R	Mild			85.9%	14.1%	88.2%	11.8%	63.3%	12.7%
	Moderate-extreme^1^			20.4%	79.6%	28.1%	71.9%	36.7%	87.3%
	Krippendorff’s α [95%-CI]^2^			0.64 [0.60; 0.67]		0.57 [0.53; 0.61]		0.48 [0.44; 0.52]	
MAQ	Mild					83.9%	16.1%	72.0%	16.2%
	Moderate-extreme^1^					24.6%	75.4%	28.0%	83.8%
	Krippendorff’s α [95%-CI]^2^					0.59 [0.55; 0.62]		0.55 [0.51; 0.58]	
MQ	Not clinically significant							76.8%	22.3%
	Clinically significant^1^							23.2%	77.7%
	Krippendorff’s α [95%-CI]^2^							0.54 [0.50; 0.58]	

A-MISO-S, Amsterdam Misophonia Scale; AMISOS-R, Amsterdam Misophonia Scale Revised; MAQ, Misophonia Assessment Questionnaire; MQ, Misophonia Questionnaire; WSAS, Work and Social Adjustment Scale. ^1^Cutoffs for “moderate - extreme” or “clinically significant”: sumscore of 10 for A-MISO-S, of 21 for AMISOS-R, of 22 for MAQ, score of 7 in MQ item 18, c.f. Table 1. ^2^Chance-corrected agreement with 95% confidence interval derived from bootstrapping with 10,000 samples.

### 3.2. Phenomenology of individuals with misophonia with or without functional impairment

According to the WSAS, about 55% of the participants experienced no or mild and 45% experienced significant functional impairment *due to* misophonia symptoms (with 42.8% moderate, 1.8% severe impairment). Table 2 shows concordance rates and Krippendorff’s α for the WSAS with the binary misophonia questionnaires. Means and standard deviations on misophonic symptom severity, other ICD-10 related psychopathology symptoms, interpersonal emotion regulation, and quality of life separated by group (functional impairment yes/no) are presented in Table 3. T-tests on the non-misophonia measures revealed significant differences between the groups regarding psychopathology symptom severity, *t* (1512.68) = −18.43, *p* < 0.001, *d* = 0.88, and quality of life, *t* (1813) = 11.78, *p* < 0.001, *d* = 0.56, indicating higher psychopathology and lower quality of life in the group with versus without functional impairing misophonia symptoms. Significant group differences on interpersonal emotion regulation scores were found for the subscales *enhancing positive affect*, *t* (1853) = 4.45, *p* < 0.001, *d* = 0.21, *perspective taking*: *t* (1852) = 3.79, *p* < 0.001, *d* = 0.18, and *soothing*, *t* (1855) = 4.68, *p* < 0.001, *d* = 0.22, indicating that individuals with versus without functional impairing misophonia use these emotion regulation strategies less often. No significant group differences were found regarding the subscale *social modeling*, *t* (1849) = 1.37, *p* = 0.17, *d* = 0.06. Frequencies of potential self-reported comorbid and/or differential diagnoses or phenomena ranged from 0.7% (phonophobia) to 30.5% (ASMR). The frequencies separated per functional impairment due to misophonia are presented in Table 3, revealing significantly higher rates of phonophobia, hyperacusis, sensory processing disorder or hypersensitivity, and mental disorders in the group with versus without functional impairments due to misophonia. The odds of a phonophobia or hyperacusis diagnosis were around 7 times higher with versus without functional impairments due to misophonia, and around 1.8 times higher for sensory processing disorder/hypersensitivity or a diagnosed mental disorder.

**Table 3:** Misophonia symptom severity and other characteristics in individuals without (*n* = 1,042) and with (*n* = 839) functional impairing misophonia.

		No misophonia or without functional impairment^1^	Misophonia with functional impairment	Test statistics^2^		Subgroups of functional impairing misophonia - severity	
						Moderate	Severe
						*n* = 805	*n* = 34
Questionnaire	Possible range	*M* (*SD*)/% (*n*)	*M* (*SD*)/% (*n*)	*χ*^2^ (1)	Odds ratio	*M* (*SD*)	*M* (*SD*)
Amsterdam Misophonia Scale, A-Miso-S	0–24	7.66 (3.95)	13.22 (3.39)	-	-	13.04 (3.27)	18.03 (2.75)
Amsterdam Misophonia Scale Revised, AMISOS-R	0–40	16.97 (8.32)	27.15 (5.92)	-	-	26.88 (5.82)	34.03 (3.86)
Misophonia Assessment Questionnaire, MAQ	0–63	14.42 (11.91)	34.11 (12.06)	-	-	33.52 (11.73)	49.10 (10.86)
Misophonia Questionnaire, MQ	0–68	23.24 (10.56)	36.27 (8.16)	-	-	35.90 (7.97)	45.57 (7.32)
Misophonia Screening List, MSL	0–56	21.08 (12.86)	38.21 (9.75)	-	-	37.82 (9.62)	48.17 (7.42)
Diagnosed phonophobia	n/a	0.2 (2)	1.3 (11)	8.57, *p* = 0.004	6.96 [1.99; 20.11]		
Diagnosed hyperacusis	n/a	0.4 (7)	1.9 (36)	27.54, *p* < 0.001	6.68 [2.96; 15.10]		
Diagnosed tinnitus	n/a	6.5 (119)	5.2 (96)	0.01, *p* = 0.944	1.01 [0.77; 1.37]		
Diagnosed audiological/ hearing disorders	n/a	6.7 (123)	6.0 (110)	0.84, *p* = 0.397	1.14 [0.88; 1.48]		
Sensory processing disorders or hypersensitivity	n/a	1.9 (35)	2.7 (50)	7.48, *p* = 0.007	1.84 [1.19; 2.99]		
ASMR	n/a	16.7 (308)	13.8 (254)	0.19, *p* = 0.684	1.05 [0.86; 1.28]		
Synesthesia	n/a	5.1 (94)	4.7 (86)	0.91, *p* = 0.344	1.16 [0.86; 1.59]		
Diagnosed mental disorder	n/a	13.5 (248)	16.0 (294)	30.00, *p* < 0.001	1.75 [1.43; 2.15]		
ICD-10 Symptom rating (ISR)	0–4	0.66 (0.50)	1.16 (0.66)***			1.13 (0.63)	1.97 (0.76)
Quality of life - BIFL	−15–15	3.62 (4.01)	1.40 (3.90)***			1.50 (3.91)	−1.16 (2.57)
IERQ: Enhancing positive affect	5–25	17.64 (4.44)	16.71 (4.55)***			16.75 (4.54)	15.63 (4.59)
IERQ: Perspective taking	5–25	11.66 (3.95)	10.91 (3.84)***			10.93 (3.84)	10.53 (3.92)
IERQ: Soothing	5–25	13.06 (4.48)	12.11 (4.44)***			12.11 (4.43)	12.17 (4.74)
IERQ: Social modeling	5–25	14.64 (4.61)	14.34 (4.60), n.s.			14.34 (4.59)	14.30 (5.00)

^1^Based on misophonia-specific functional impairment according to the WSAS: Work and Social Adjustment Scale. ^2^The other test statistics are presented in the main text. IERQ: Interpersonal Emotion Regulation Questionnaire, BIFL: Bielefelder Questionnaire on Quality of Life. Higher values indicate higher symptom severity, more adaptive emotion regulation (IERQ) and higher quality of life (BIFL), respectively. ***: *p* < 0.001. No significance testing was performed for the misophonia measures and between the clinical subgroups. ASMR: Autonomous Sensory Meridian Response.

Regarding the Misophonia Trigger list (MT), the ten most annoying triggers were all sound triggers and almost identical in individuals with versus without clinical misophonia (see Table 4). Most sounds were related to eating (60% in both groups) and breathing (30% in the clinical, 20% in the non−/subclinical-group). Only 10% (20%) were unrelated to eating or breathing. In the group of the 235 individuals (12.5%), who indicated no sound sensitivity at all in the first item of the survey, 50% of the ten most annoying triggers still matched to the list in Table 4. The other 50% of potential triggers included more general unpleasant sounds (i.e., jackhammer, dentist drill, a baby crying, many people talking at the same time).

**Table 4:** Ten most annoying triggers.

	Misophonia with functional impairment *n* = 839	No misophonia or without functional impairment *n* = 1,042
Trigger	Rank	*M*	Rank	*M*
Smacking	1	3.59	1	2.89
Slurping	2	3.23	4	2.28
Chewing	3	3.18	3	2.37
Snoring	4	3.11	2	2.47
Talking with mouth full	5	3.03	6	2.14
Fork scraping along teeth	6	2.82	7	2.01
Nose whistling	7	2.81	9	1.84
Swallowing	8	2.68	10	1.81
Nasal breathing	9	2.54	–	–
Chalk squeaking on a blackboard	10	2.49	5	2.17
Mosquito buzzing	–	–	8	1.87

Level of annoyance of seventy-nine triggers was rated with the Misophonia Trigger List (MT), range 0–4.

Individuals in the clinical (*M* = 2.95, *SD* = 0.34) versus non−/subclinical group (*M* = 2.18, *SD* = 0.34) rated the level of annoyance caused by the triggers as significantly higher, *t* (1879) = 48.42, *p* < 0.001, *d* = 2.26, and they rated significantly more triggers as at least slightly annoying (*M* = 50.60, *SD* = 14.90 vs. *M* = 35.45, *SD* = 16.00), *t* (1839.62) = −21.20, *p* < 0.001, *d* = 0.98.

**Figure 1:**
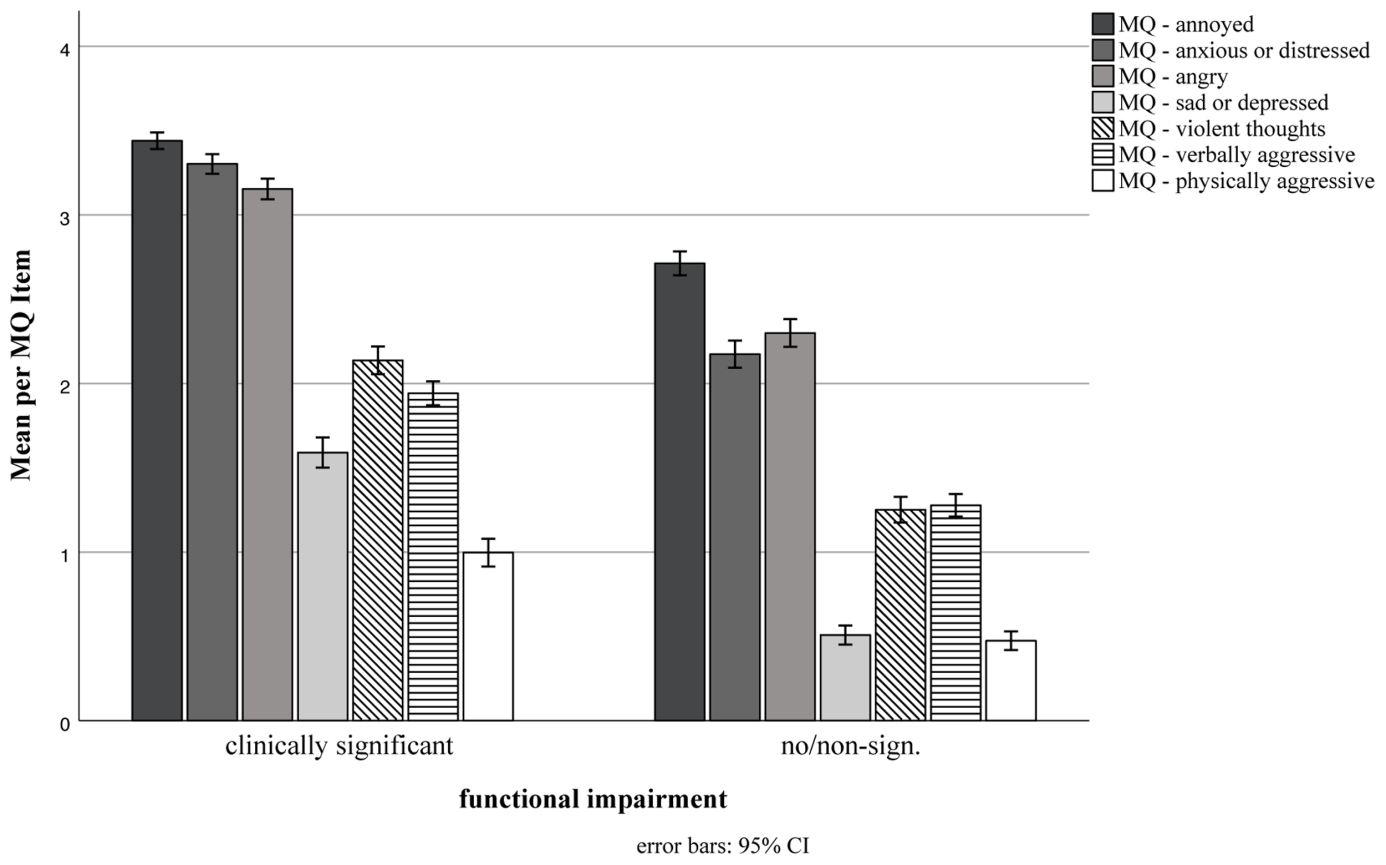
Emotional and behavioral reactions to trigger. Groups based on misophonia-specific functional impairment according to the WSAS: Work and Social Adjustment Scale. Items of the Misophonia Questionnaire (MQ) range from 0 “never” to 4 “always.” 95% CI = 95% confidence interval.

The most frequent emotional and behavioral reactions toward triggers (MQ) per group are depicted in Figure 1. Similar rank orders across the different reactions were observed between groups. On a descriptive level, item mean differences between groups for emotional reactions were highest for anxious/distressed reactions (clinical group: *M* = 3.3, *SD* = 0.86; non−/subclinical: *M* = 2.17, *SD* = 1.33), followed by sad/depressed reactions (clinical group: *M* = 1.59, *SD* = 1.32; non−/subclinical: *M* = 0.51, *SD* = 0.92), see Figure 1. In the AMISOS-R, emotional reactions (irritation, anger, disgust, and other) are either affirmed or denied. The most frequently affirmed reaction was irritation (94% vs. 88% in the clinically impaired vs. non-impaired group), followed by anger (80% vs. 55%), and disgust (42% vs. 30%).

### 3.3. Recommended cutoffs for identifying (clinically relevant) misophonia and distinguishing from no or subclinical misophonic reactions

The results of the ROC analyses are presented in Table 5 with area under the curve values ranging from 0.81 (MQ subscale) to 0.87 (MAQ).

**Table 5:** Area under the curve (AUC) and suggested cutoffs based on functional impairment due to misophonia symptoms.

Questionnaire	Possible range	Existing categorization	AUC 95% CI	Cutoffs^1^	Sensitivity 95% CI	Specificity 95% CI	Youden Index	LR +95% CI	LR−95% CI
Amsterdam Misophonia Scale, A-Miso-S	0–24	No/subclinical: 0–4 Mild: 5–9 Moderate: 10–14 Severe: 15–19 Extreme: 20–24	0.86 [0.84; 0.87]	10	0.89 [0.86; 0.91]	0.67 [0.64; 0.70]	0.55	2.65 [2.47; 2.97]	0.17 [0.13; 0.20]
Amsterdam Misophonia Scale Revised, AMISOS-R	0–40	No/subclinical: 0–10 Mild:11–20 Moderate severe: 21–30 Severe to extreme: 31–40	0.84 [0.82; 0.86]	2324	0.80 [0.78; 0.83] 0.76 [0.72; 0.78]	0.72 [0.69; 0.75] 0.76 [0.74; 0.79]	0.52	2.85 [2.58; 3.19] 3.20 [2.84; 3.59]	0.27 [0.24; 0.32] 0.32 [0.28; 0.36]
Misophonia Assessment Questionnaire, MAQ	0–63	Mild: 0–21 Moderate: 22–42 Severe: 43–63	0.87 [0.85; 0.89]	24	0.81 [0.78; 0.84]	0.77 [0.75; 0.80]	0.58	3.53 [3.14; 3.98]	0.25 [0.21; 0.28]
Misophonia questionnaire, MQ	0–68	n/a	0.83 [0.82; 0.85]	30 31	0.80 [0.77; 0.83] 0.76 [0.73; 0.79]	0.70 [0.67; 0.72] 0.74 [0.71; 0.77]	0.50	2.65 [2.42; 2.96] 2.91 [2.63; 3.29]	0.28 [0.25; 0.33] 0.32 [0.28; 0.37]
MQ item 18	0–15	clinically significant: > 6	0.84 [0.82; 0.85]	7	0.78 [0.75; 0.80]	0.77 [0.74; 0.79]	0.54	3.31 [3.03; 3.84]	0.29 [0.25; 0.33]
MQ subscale emotions and behaviors	0–50	n/a	0.81 [0.80; 0.83]	19 20 21	0.81 [0.79; 0.84] 0.76 [0.73; 0.79] 0.70 [0.67; 0.73]	0.65 [0.62; 0.68] 0.70 [0.67; 0.73] 0.76 [0.73; 0.78]	0.46	2.33 [2.12; 2.54] 2.56 [2.29; 2.81] 2.88 [2.61; 3.30]	0.29 [0.25; 0.34] 0.34 [0.30; 0.39] 0.39 [0.35; 0.44]
Misophonia screening list, MSL	0–56	n/a	0.85 [0.83; 0.87]	31	0.80 [0.77; 0.82]	0.74 [0.71; 0.76]	0.54	3.03 [2.77; 3.46]	0.27 [0.23; 0.31]

95% CI: bootstrapped 95% confidence intervals, LR+ and LI−: positive and negative diagnostic likelihood ratios (LR+ = sensitivity/(1-specificity); LR− = (1-sensitivity)/specificity). ^1^The cutoffs are decimal rounded and defined as greater than and equal to the respective values (≥). The presented cutoffs per measure have the same (maximum) Youden index (the sum of sensitivity and 1-specificity).

## 4. Discussion

The present study aimed at assessing the frequency of misophonia symptom (severity) in a large non-representative sample according to common misophonia measures, describing differences in the clinical picture of misophonia for those with versus without significant functional impairment, and to provide cutoffs on misophonia questionnaires for the purpose of identifying those best who reported significant functional impairment in their daily lives due to misophonia, and as such, are most likely individuals who would need access to and benefit from treatment for this condition.

First of all, this is to our knowledge the first study to compare scoring across various misophonia symptom measures in a large sample of *n* = 1,881. We found 85% of the individuals in the mild-to-severe misophonia symptoms range based on the A-MISO-S or AMISOS-R. The MQ and MAQ only identified 50% of this sample to be in the clinical range. The measure comparisons revealed that 12 to 28% of individuals scored in the “mild group” of one but the “moderate–severe” group of another misophonia measure, indicating that the measures at least partly assess different aspects of misophonia. The pattern was even more pronounced for the functional impairment measure WSAS compared to symptom severity measures, with a proportion of up to 37%. This underlines the subordinate role of misophonia-caused functional impairment in the classification into mild and moderate–severe of these measures. It demonstrates that the majority of classifications within misophonia measures so far are based primarily on severity (e.g., simply reflecting the extent to which a person is triggered by sounds) and put less emphasis on the difference between impairment due to and severity of the misophonic reaction. The consequences may be less significant the more severe the misophonic reaction, as with increasing severity is it likely to also find more impairment. However, especially in the moderate range of misophonic reactions, this discrimination may be significant. The developers of the A-MISO-S and AMISOS-R, respectively, name the lowest symptom class “subclinical,” implying that all other symptom classes exceed the clinical threshold: the mild symptom class and the moderate and severe class (18, 29). Based on our results, we suggest to conceptualize the mild symptom class still as subclinical, rather reflecting variations in this specific form of decreased sound tolerance than meeting threshold for a clinical disorder. This would also be in line with our attempt to identify a disorder (clinical threshold) by using misophonic-related functional impairment in daily lives as a quantification of one of the suggested revised diagnostic criteria for misophonia (19). When differentiating the sample in those reporting clinically significant impairment, we identified 44% of the present sample to be affected by a clinical expression of misophonia. This is much lower than the number of participants who self-assigned to the condition before they started the survey (87.5%). Similarly, Williams et al. identified 7.3% as clinical and 10% as subclinical misophonia cases using the DVMSQ that differentiated between the groups by a functional impairment item (15). On the one hand, these findings demonstrate that experiencing symptoms, such as not liking specific sound stimuli, is overestimating the presence of a functional impairing condition. On the other hand, it underlines that self-identification with misophonia is rather based on a dimensional construct than a categorical disorder approach. We follow up on a suggestion made by a reviewer of this article (Zack Williams) and propose to use the term “misophonic disorder” for a clinically relevant manifestation of this condition only and use “misophonic reactions” when subclinical manifestations are the focus of research on misophonia. Thus, we suggest misophonia might be used as an umbrella term for all manifestations, analog to for example, depression for depressive symptoms and depressive disorders or in the context of tinnitus symptoms (and tinnitus as a disorder) (47). The dimensional approach still leaves open what defines the respective underlying construct (s). We propose to follow up on this aspect in future research investigating whether the tendency of experiencing misophonic (emotional) reactions varies between individuals; and if this individual tendency is rather representing a risk factor for the development of a misophonic disorder (alike behavioral inhibition in anxiety disorders) or if it is in itself a main criterion of the disorder and only needs to reach a certain threshold. For example, it might be important to compare the patterns of symptoms in non-misophonic, subclinical, and clinical misophonia groups across multidimensional measures.

We determined large group differences between the subclinical and the clinical manifestation with literally more severe/more frequent symptom scores across all measures, independently of whether they focus on misophonic symptoms or general psychopathology including depressive, anxiety, or obsessive–compulsive symptoms. These differences also extend to other concepts, such as quality of life with medium and interpersonal emotion regulation skills with small effect sizes. This study is adding a comparison group study to the existing evidence and it suggests mostly more severe reactions in the clinical compared to the non−/subclinical group (16, 19) and extends findings on emotion regulation (22) to interpersonal emotion regulation skills. However, as in (48), there is also evidence for qualitative differences, e.g., in emotional reactions to misophonic stimuli.

Regarding potential comorbid or differential diagnoses and phenomena, the pattern is similar for diagnosed hyperacusis and phonophobia with odds around 7 times higher for these diagnoses in the clinical versus non−/subclinical group. However, the rates of diagnosed tinnitus and experiences of ASMR and synesthesia were not significantly different and reported less often in the group with clinical versus non−/subclinical manifestations of misophonia. Finally, the types of stimuli that are experienced as annoying are vastly the same across both groups, primarily sounds related to eating and breathing. This may indicate a normative power of these stimuli to elicit annoyance in many individuals, although this should be interpreted with caution in light of the high proportion of individuals self-identifying with misophonia in the current study.

However, it remains unclear why these sounds are annoying for so many people. The similarity in trigger sounds in both groups may imply cognitive and emotional processes involved in evaluating the stimuli to be key when trying to make a difference for those affected by misophonia (23). In the current study, the difference between functionally impaired and non-impaired groups rather lay in the intensity to which these stimuli were experienced and evaluated as annoying. In line with the idea mentioned earlier, we hypothesize that the difference between clinical and subclinical manifestations may be more a matter of quantity than quality (“quantity hypothesis”); that is a clinical group may not be characterized by a specific symptom profile or different (types of) stimuli that function as a trigger but may be more reflected by similar symptoms and a similar ranking of functional stimuli (i.e., with the power to trigger the specific emotional, behavioral and physiological reactions associated with misophonia). Similarly, Savard et al. (49) found support for the idea of a continuum for misophonia symptom severity from their online-survey results. Additionally, derived from results of an experimental masking paradigm with different (trigger) sounds, they found that individuals with high versus low misophonia symptoms had similar detection rates of sounds—triggers were identified best in both groups, followed by unpleasant and neutral sounds—but differed regarding the subsequent emotional reaction with higher ratings of negative emotions in the high versus low symptom group.

Interestingly, the “quantity hypothesis” appears to also apply within the clinical group: when examining differences between moderate and severe clinical presentations, all scores descriptively increase when moving to the severe group. The size of these effects varies around one standard deviation (*d* = 0.69–1.53), roughly, across all measures—except for the interpersonal emotion regulation questionnaire. Effect sizes for the differences drop clearly with the largest one reaching roughly *d* = 0.25 on “Enhancing positive affect,” and going further down to *d* < 0.01 on the other three scales. Thus, the differences on the interpersonal emotion regulation subscales are statistically significant (exception: social modeling). However, the effects are small and were only detectable due to the large sample size, which may suggest that the group differences for the subscales “soothing” and “perspective taking” are not clinically meaningful. At the same time, this may point at the relative significance of positive emotion regulation and the lack of adaptive regulation skills within the clinical group while both interpersonal maladaptive and adaptive emotion regulation skills distinguish clinical from subclinical presentations. The interpersonal emotion regulation questionnaire is a measure that reflects how individuals may utilize others to regulate their own emotions (41) and this specific subscale “Enhancing positive affect” is significantly correlated with reappraisal, tolerating and accepting emotion regulation skills. It may be beneficial to include a treatment module on how to deliberately improve one’s own positive affect when working with more severe cases (in addition to working on reducing maladaptive interpersonal emotion regulation for everybody in treatment). Another interpretation of these findings would be that the lack of interpersonal emotion regulation is adaptive in the clinical group as long as misophonia symptoms are still present, as individuals with misophonia may have experienced invalidation of their emotions and others might rather trigger (negative) emotions than regulate them. These suggestions need to be considered with caution because the comparisons rely on very different sample sizes per group. The percentage of those with severe presentations is proportionally small in the entire sample. Nevertheless, it is still a considerable number of individuals (*n* = 34) and in light of the few empirical findings helping to derive treatment recommendations, it may add some further ideas on what to include in psychological treatment (packages). Further, we mainly looked at interpersonal enhancing positive affect. This does not necessarily relate to one’s own skills. When thinking about clinical implications, one way to go may include a module of specific emotion regulation skills. However, it is unclear from our results, if individuals are generally lacking in their ability to experience positive affect or if this is more specific to interpersonal emotion regulation. In the latter case, it may also imply to include interventions related to drawing on others to support their own emotion regulation. Following the interpretation of the lack of interpersonal emotion regulation as adaptive and a result of misophonia, interpersonal emotion regulation would not be targeted in treatment but would possibly improve eventually with a decrease in misophonia symptoms. These different implications may be tested in future studies.

Another interesting result relates to the emotions experienced in response to triggers. It may be noted that misophonia measures partly differ regarding the emotional reactions they assess. While the A-MISO-S and AMISOS-R do not assess anxious/distressed reactions—in contrast to the MQ—the latter does not assess disgust. According to the MQ, annoying, anxious/distressed, and angry emotional responses to misophonic stimuli were the responses most frequently endorsed in our study. Although sad/depressed feelings also occur (similarly disgust, AMISOS-R), they ranked clearly behind anxiety and anger, mentioned only about half as often. Comparing irritation and anger, group differences in endorsement rates were larger for anger, indicating that anger might be better able to discriminate clinical from subclinical reactions. In the consensus definition of misophonia, Swedo et al. (1) name all the aforementioned emotions as “most common” reactions to triggers (pp. 10) and dispense with weighting further. In contrast, Jager et al. (19) define (only) irritation, anger and/or disgust as required emotional reaction toward triggers in their revised criteria of misophonia, and leave out anxiety. They discuss anxiety as potentially mistaken emotional response as their study participants with clinical misophonia did not mention anxiety as first (i.e., as fear reaction) but either anticipatory or secondary emotional response. Similarly, lower endorsement rates of the fear/panic item were found in the validation of the Duke-Vanderbilt Misophonia Screening Questionnaire (DVMSQ) (15). As distress and anxiety are combined in the MQ item, they cannot be disentangled in the responses. Because of the somewhat mixed findings across studies, we suggest continuing to capture all these different emotions, including disgust and anxiety, as a potential response in research studies until the evidence basis points more clearly to an emotional reaction which is the most typical and/or differentiates misophonic disorder best from subclinical misophonic reactions or other conditions.

Regarding group differences in the current study, with severity increasing, the steepest increase is observed in anxious or depressed emotional responses relative to angry and annoyed responses when exposed to annoying stimuli. Externalizing attributions may increase the externalizing emotional reactions (i.e., angry, annoyed). We speculate that this externalizing pattern might be common along the continuum of symptom severity, whereas the internalizing emotional reactions might appear more often and more strongly in those individuals with higher, clinical symptom severity and duration. Thus, providing skills to regulate specifically anger and/or attributional retraining may be another promising candidate for a treatment module—on the one hand, to prevent the significantly increased occurrence of verbally as well as physically aggressive reactions in individuals with clinical misophonia (medium effect sizes) and, on the other hand, to prevent or decrease secondary depressive reactions.

Finally, when screening for misophonia with the purpose of identifying clinical manifestations that deserve treatment-related attention, it is recommended to use a higher cutoff on the common instruments for misophonia symptoms (e.g., A-MISO-S or AMISO-R) or to add a functional impairment requirement in the interpretation of the scales. A (mental) disorder usually requires clear impairment in areas of daily life (e.g., ICD-11) (2) and health insurances usually only cover treatment expenses when a clinical threshold is met. While this diagnostic issue is best addressed with a clinician-administered interview, pre-selection, for example for inclusion in randomized controlled treatment trials, could be successfully conducted based on recommended cutoff scores. We provide several possibilities of sum score cutoffs across misophonia instruments with associated costs and benefits on sensitivity and specificity to help tailoring the cutoff score to the associated research purpose^2^. Of those published previously (e.g., AMISO-S, AMISO-R), our recommended cutoffs are higher, and of those without cutoff recommendations so far (MQ, MAQ), we provided our suggestions. The former indicates that clinical cases may have been underrepresented in previous studies, specifically in the mild symptom class.

Assignment to a class also requires reflecting upon the best conceptual match with other examples of the overall disorder group. Focusing on the emotional reactions, one might come to different conclusions depending on which emotion (s) one focuses on. Whereas anger, irritation, or fear/anxiety indicate an emotional disorder (anxiety and mood disorders, e.g., disruptive mood dysregulation disorder), distress and/or disgust reactions point more toward an obsessive–compulsive spectrum. Even if there is rather little evidence from our study that OCD-related assumptions maybe transferred to this condition, valid conclusions still appear premature. Progress in classification also requires to reflect upon potentially effective psychological treatments. The present study supports CBT strategies already used in the treatment of misophonia such as attention training and arousal reduction (51). Further, it suggests that it may be worthwhile to assess changes in interpersonal emotion regulation in other interpersonal contexts beyond the misophonic reaction itself during treatment. If individuals experience persisting difficulties, the therapeutic strategies may be extended to interpersonal emotion regulation, for example, teaching (specific) emotion regulation skills to enhance positive affect as well as skills to reduce negative affect and anger in these non-misophonic contexts in general.

### 4.1. Limitations

Compared to the general population, a disproportionate number of female, highly educated, young individuals participated in the study. This reflects a typical but not less critical pattern, which should be addressed with targeted sampling in future studies to improve generalizability. Similarly, (online) surveys that disclose misophonia as a central topic, lead to inclusion of more individuals with an increased interest in the topic and/or self-identifying with the condition, which does not allow conclusions about the prevalence of misophonia in general and may also be a potential source of bias regarding other results. However, the self-selection by interest and subjective affectedness also led to many participants with symptom severity around our diagnostic threshold so we could compare and differentiate the two groups particularly well. It should be noted that we did not include attention checks in the survey and that there was no compensation for every participant. However, we did check if we detect unusual response patterns (e.g., random or careless responses to the survey questions) and did not find evidence for any clear pattern in the participants. Some of the misophonia measures were primarily developed to assess symptom severity in individuals with (probable) misophonia and may thus include terms which are more intuitively comprehensible by individuals familiar with the phenomenon. Another limitation results from the use of self-report measures only without a diagnostic interview and without comprehensively addressing other forms of decreased sound tolerance which would allow the identification of differential diagnoses and/or comorbidities. The selection of the five misophonia questionnaires used in the current study was based rather on the level of dissemination at the time of data collection and does not reflect all available misophonia questionnaires. Other measures which were developed or published afterward could naturally not be considered.

### 4.2. Conclusion

In the present study, a large difference of roughly 45% was found between the proportion of individuals who indicated the description of misophonia as applying to them (87.5%) and those who revealed functional impairments due to misophonia symptoms (44%). We suggest to use the latter as a criterion to differentiate individuals with (subclinical) misophonic reactions from those with misophonic disorder. Accordingly, we provide cutoffs for five different misophonia measures (A-MISO-S, AMISOS-R, MSL, MQ, and MAQ) which best detect individuals with and without misophonic disorder (i.e., with and without functional impairments due to misophonic reactions) in settings in which a diagnostic interview is not applicable. These values partly differ from existing classifications of some of the measures in that they raise the threshold for clinical misophonia.

The comparison of the groups regarding aspects of misophonia and other characteristics, such as general psychopathology or quality of life, revealed rather quantitative differences, such as increasing symptom severity or number of triggers with higher functional impairments. However, some misophonic reactions and associated aspects appeared more typical—in both groups. For example, annoyed and anxious/distressed reactions were experienced more frequently compared to reactions of disgust and sadness/depression. Similarly, within interpersonal emotion regulation skills, especially skills to enhance positive affect were significantly impaired with increasing misophonia severity. We suggest to include and extend specific interventions on improving the regulation of negative, more externalizing emotions, as the typical misophonic reactions and also target the ability to enhance positive affect.

## Data availability statement

The raw data supporting the conclusions of this article will be made available by the authors, without undue reservation.

## Ethics statement

The studies involving human participants were reviewed and approved by ethics committee, Bielefeld University, Bielefeld, Germany. The patients/participants provided their written informed consent to participate in this study.

## Author contributions

AM: formal analysis, data curation, writing—original, and writing—review and editing. NH: conceptualization, resources, writing—original, writing—review and editing, and supervision. LI: investigation, project administration, writing—review and editing. NP: investigation, project administration, and writing—review and editing. HK: conceptualization, investigation, resources, writing—review and editing, and supervision. All authors contributed to the article and approved the submitted version.

## Funding

We acknowledge the financial support of the German Research Foundation (DFG) and the Open Access Publication Fund of Bielefeld University for the article processing charge.

## Conflict of interest

The authors declare that the research was conducted in the absence of any commercial or financial relationships that could be construed as a potential conflict of interest.

## Publisher’s note

All claims expressed in this article are solely those of the authors and do not necessarily represent those of their affiliated organizations, or those of the publisher, the editors and the reviewers. Any product that may be evaluated in this article, or claim that may be made by its manufacturer, is not guaranteed or endorsed by the publisher.

## Footnotes

^1^The data will be made available by the first author upon request *via* e-mail.

^2^We provide tables with sensitivity and specificity per sum score per measure *via* the project on the Open Science Framework repository (50) to enable a selection of an optimal cutoff depending on the purpose.
