# Supplementary material for: The central role of symptom severity and associated characteristics for functional impairment in misophonia
Source: Front Psychiatry. 2023 Mar 28;14:1112472. doi: 10.3389/fpsyt.2023.1112472 (PMC10086372; doi:10.3389/fpsyt.2023.1112472)
Supplement: Supplementary file 1 [file Data_Sheet_1.PDF]

ESM1 – Information on translation process of Misophonia measures

1 Basic information

All measures used in the study have been translated to German with permission of the authors, according to the following protocol[1]:

1) Translation original to German by a professional translator
2) Discussion in and adaptations by German expert panel (i.e. licensed psychotherapists, experienced in diagnosing and treating patients with misophonia)
3) Backtranslation German to original by independent professional or mother tongue translator
4) Discussion of differences between translator, German expert panel and/or authors of the original version. Adaptation of German version, if needed.
5) Finalizing of German version

[1] Adapted from a World Health Organization translation protocol, WHODAS 2.0 Translation guidelines.pdf. https://terrance.who.int/mediacentre/data/WHODAS/Guidelines/WHODAS%202.0%20Translation%20guidelines.pdf [Accessed November 29, 2022]

2 Overview of measures

Measure		Original		German Version	
**Name**	**Short**	**Cited in manuscript as**	**Language**	**Permission to develop German version**	**Cited in manuscript as**
Amsterdam Misophonia Scale	A-Miso-S	Schröder A, Vulink N, Denys D. Misophonia: Diagnostic Criteria for a New Psychiatric Disorder. *PLoS ONE* (2013) doi: 10.1371/journal.pone.0054706	Dutch	By Arjan Schröder in July 2020 (From English Version)	Potthast N, Illies L, Kley H. Amsterdam Misophonia Scale - German translation. (2020)
Amsterdam Misophonia Scale Revised	AMISOS-R	Schröder A, Spape M. Amsterdam misophonia scale – revised (AMISOS-R). *Unpubl Instrum* (2014) Retrieved from https://journals.plos.org/plosone/article?id=10.1371/journal.pone.0231390#sec007	Dutch	By Inge Jager, Damiaan Denys and Arjan Schröder in August 2020	Kley H. German translation of the Amsterdam misophonia scale – revised (AMISOS-R). (2020)
Misophonia Screening List	MSL	Schröder A, Spape M. Misophonia Screening List. *Unpubl Instrum* (2014) Retrieved from https://journals.plos.org/plosone/article?id=10.1371/journal.pone.0231390#sec007	Dutch	By Inge Jager, Damiaan Denys and Arjan Schröder in August 2020	Kley H. Misophonia Screening List - German translation. (2020)
Misophonia Assessment Questionnaire	MAQ	Johnson M. 50 cases of Misophonia using the MMP. (2014)	English	By Marsha Johnson in 2018	Potthast N, Illies L, Kley H. Misophonia Assessment Questionnaire (MAQ) - German translation. (2020)
Misophonia Questionnaire	MQ	Wu MS, Lewin AB, Murphy TK, Storch EA. Misophonia: Incidence, Phenomenology, and Clinical Correlates in an Undergraduate Student Sample. *J Clin Psychol* (2014) 70:994–1007. doi: 10.1002/jclp.22098	English	By Monica Wu in 2018	Potthast N, Illies L, Kley H. Misophonia Questionnaire (MQ) - German translation. (2020)
